# Emerging models of human and non-human primate placental development – Centre for Trophoblast Research 17th annual meeting 2024

**DOI:** 10.1242/bio.061774

**Published:** 2024-11-28

**Authors:** Irving L. M. H. Aye

**Affiliations:** ^1^ Department of Obstetrics and Gynaecology, University of Cambridge, NIHR Cambridge Comprehensive Biomedical Research Centre, Cambridge, CB2 0SW, United Kingdom; ^2^ Centre for Trophoblast Research (CTR), Department of Physiology, Development and Neuroscience, University of Cambridge, Cambridge, CB2 3EL, United Kingdom; ^3^ Wellcome – Medical Research Council Stem Cell Cambridge Stem Cell Institute (CSCI), University of Cambridge, Cambridge, CB2 0AW, United Kingdom

**Keywords:** Development, Embryo, Placenta, Pregnancy

## Abstract

The 17th annual meeting of the Centre for Trophoblast Research (CTR) took place at the University of Cambridge, UK, on 1-2 July 2024. This year's meeting provided an opportunity to reflect on the significant advancements made recently in modelling the human placenta *in vitro*. The meeting featured 12 invited speakers and attracted 260 participants from 25 countries. Many of the speakers were leading figures who have developed methods to derive human trophoblast stem cells or organoids from first trimester and term placentas, and from pluripotent stem cells. Accompanying the invited presentations were flash talks selected from the abstract submissions and poster presentations. The meeting concluded with a stimulating panel discussion to evaluate the current human trophoblast models. This Meeting Review aims to capture the spirit of the event and highlight the key themes and take-home messages that emerged.

## Teaser

**Summary:** This Meeting Review highlights the recent progress in modeling the human placenta *in vitro*, their advantages and limitations, as well as the challenges that remain this field.

## Introduction

The trophoblast originates from the outer cell layer of blastocyst-stage embryos, which later develops into the major cell types of the placenta. In humans and non-human primates, the placenta is mainly composed of the three major trophoblastic cell types. The cytotrophoblasts form the stem or progenitor population that differentiate into syncytiotrophoblasts, which carry out maternal-fetal nutrient exchange and endocrine functions, or the extravillous trophoblasts that invade into the uterus and remodel maternal spiral arteries. These cells largely develop in the first to early second trimester of pregnancy and, thus, the ability to study their development remains a significant challenge owing to ethical and technical issues with gaining access to extraembryonic tissues during this gestational stage. Nevertheless, studying trophoblast development may hold the key towards understanding placenta-related pregnancy complications such as miscarriage, pre-eclampsia and fetal growth restriction, which make a significant contribution to the maternal and perinatal deaths that account for 6-7% of all deaths globally (W.H.O., 2008).

The Centre for Trophoblast Research (CTR) in Cambridge hosts an annual meeting with themes around placental research and reproduction. This year, the theme of the meeting was ‘emerging models of human and non-human primate placental development’. This year also marks 65 years since the first *in vitro* trophoblast cell line (BeWo) was derived from a human placental choriocarcinoma (Hertz, 1959). Considerable progress has been made since then, especially in the last 10 years where various methods to derive trophoblasts from pluripotent stem cells (PSCs), including induced PSCs (iPSCs) and embryonic stem cells, were developed (Das et al., 2007; Harun et al., 2006). More recently, methodologies to establish human trophoblast stem cells and 3D trophoblast organoid cultures combined with single cell and spatial transcriptomics, and advanced imaging technologies have accelerated our understanding of early placental development (Haider et al., 2018; Okae et al., 2018; Turco et al., 2018). We were delighted that many of the pioneers in this field were able to join us as speakers at the 17th CTR annual meeting held in Churchill College at the University of Cambridge this year.

**Fig. 1.**
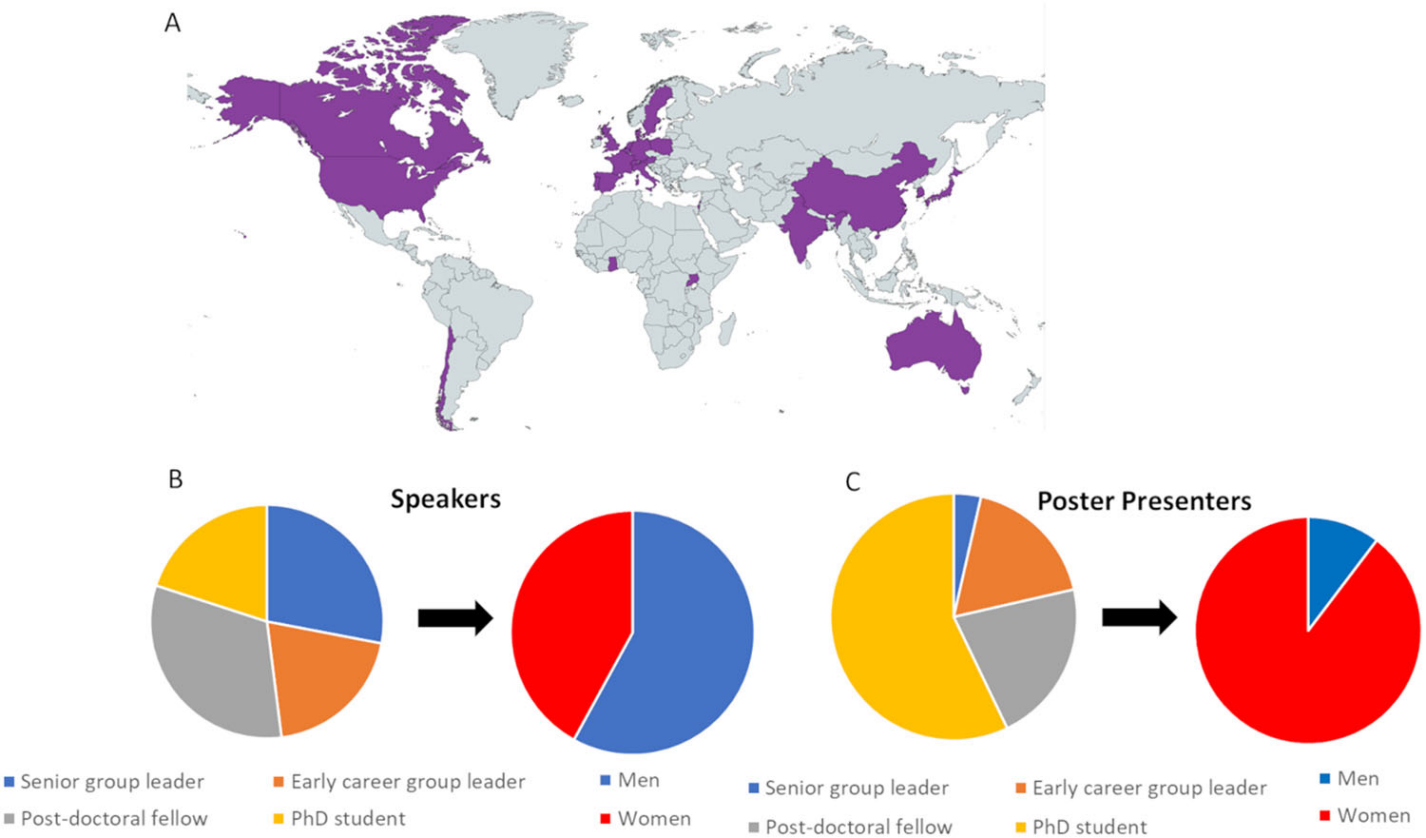
**Distribution of attendees and presenters.** (A) Distribution of attendees by the geographical location of their institutes. (B) Speakers by career stage and gender. (C) Poster presenters by career stage and gender.

When the CTR was established, one of the founding principles was to support the development of early-career researchers (ECRs). In that spirit, the CTR meeting enabled active participation and recognition of ECRs through poster presentations and flash talks selected from abstract submissions. This year, we welcomed 260 participants representing 25 countries who attended the meeting either in-person or online (Fig. 1). Most of our invited speakers had made the trip over from overseas and women made up 42% of our invited speakers. The majority of our poster presenters (22/30) were ECRs, and nearly all (90%) were women. The significant representation from ECRs highlights the importance of nurturing and supporting these emerging researchers, ensuring that the field continues to thrive and innovate.

**Fig. 2.**
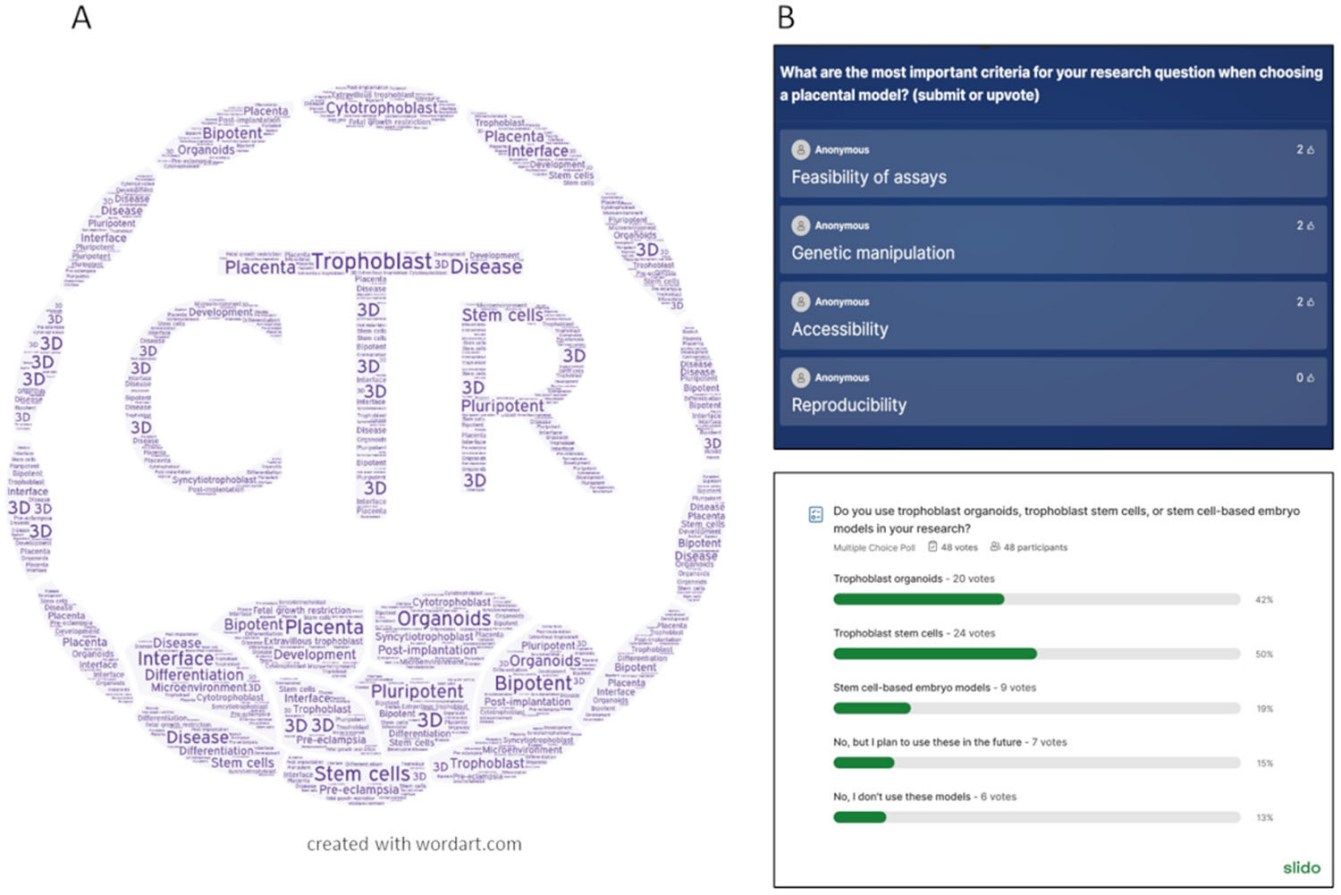
**Key themes and discussion points.** (A) A word cloud of the CTR logo representing the key themes of the meeting. (B) Interactive Q&A activities on Slido.

The hybrid format of the meeting enabled 85 participants from 17 countries to join virtually, including those from low- and middle-income countries (LMICs). The virtual component of this hybrid meeting was made possible thanks to a professional audio-visual team, enabled by a generous The Company of Biologists Sustainable Conferencing and Scientific Meetings grant. Through the online platforms Zoom and Slido, online participants were able to post their questions to the speakers and engage with the virtual attendees through live polls and Q&As in real time (Fig. 2).

**Fig. 3.**
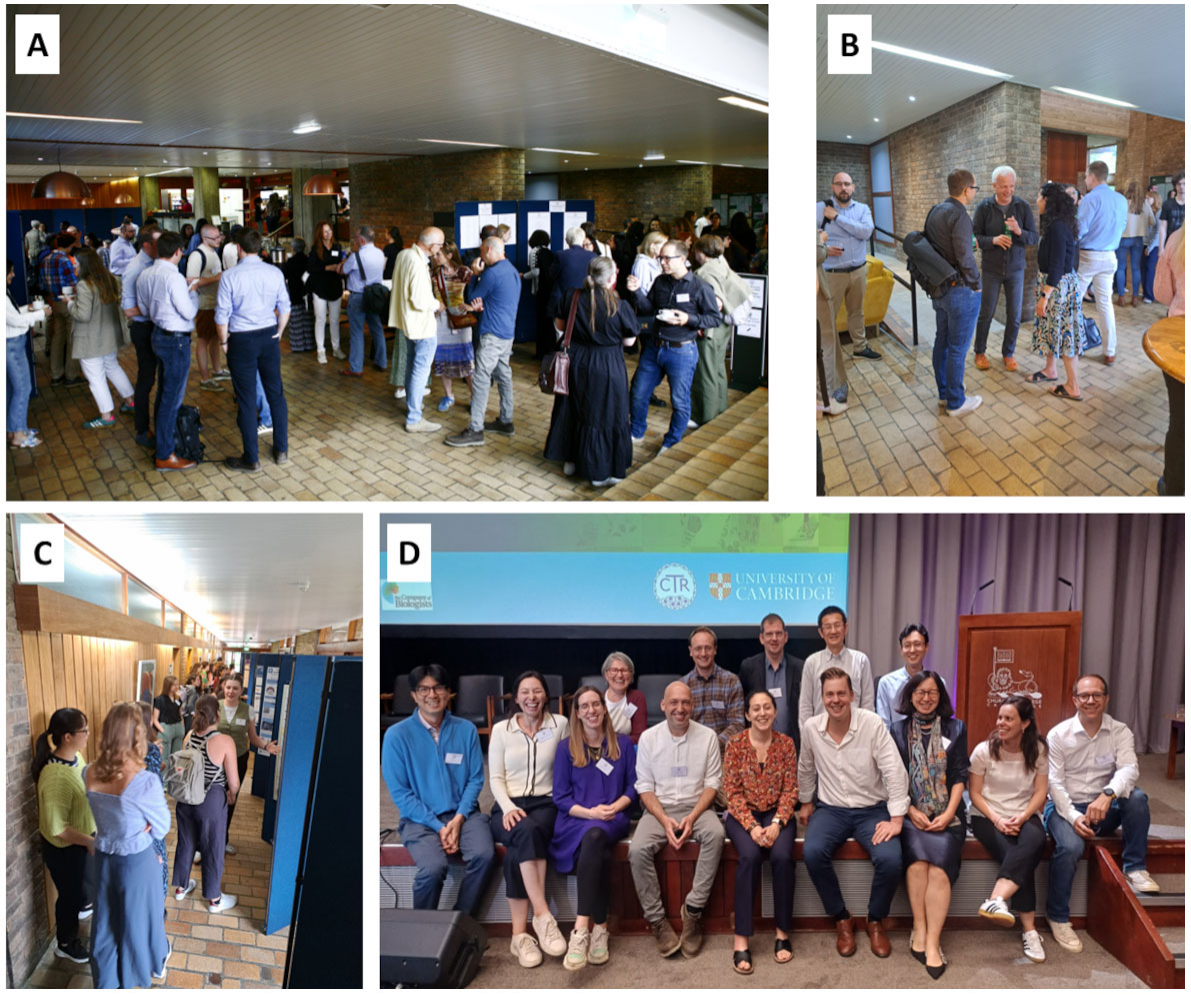
**Attendees at the CTR annual meeting 2024.** (A-C) Attendees during breaks and poster sessions. (D) Invited speakers and chairs of the meeting with the CTR Director. Photographs provided by Timo Kohler and Busma Butt

## Key themes of the meeting

The first session of the meeting centred on the latest developments in human trophoblast stem cell models. Dr Mana Parast from University of California San Diego, USA, started the session by providing an overview of the state-of-the-art *in vitro* models to study early human placental development, such as the human trophoblast stem cells (hTSCs), trophoblast organoids (TOrg) and trophoblasts differentiated from iPSCs. In addition to the value of such models, she described some of the challenges that remain including the extent to which these models reflect the *in vivo* state, and the need to benchmark these models against placental tissues wherever possible (Morey et al., 2024 preprint). To address some of these limitations, her lab has developed protocols to derive trophoblasts from primed human pluripotent stem cells to study pregnancy diseases and compared these models with primary cytotrophoblasts of different gestational ages (Soncin et al., 2022). The use of hPSCs also allows for comparison of normal versus disease states such as preeclampsia. She emphasised the need to raise awareness and educate the public to this research and advocate for sensible regulation and increased funding. Lastly, she stated the disparity in terms of ethical considerations and mandates that preclude the use of embryonic and first trimester tissues and cells in research in certain regions. The latter point is important for the placental research community as it highlights regulatory restrictions that play major roles in the type of trophoblast platforms that can be used in research.

A breakthrough in our ability to model early human placental development came in 2018 with the establishment of hTSCs by Dr Hiroaki Okae and colleagues. In the next session, Dr Okae (Kumamoto University, Japan) described his earlier work in deriving hTSCs from human first trimester placentas and blastocysts (Okae et al., 2018). He illustrated the versatility of this platform by describing his lab's recent work applying genome editing technology to hTSCs (Kobayashi et al., 2022; Takahashi et al., 2019). Lastly, he described his latest work in deriving bipotent hTSCs from term placentas by overcoming the epigenetic barriers imposed with gestational age. 2018 also marked another important milestone in trophoblast research with the publication of 3D trophoblast organoid models that recapitulate the structure and function of the chorionic villi. Margherita Turco (Friedrich Miescher Institute for Biomedical Research, Switzerland) was one of the first to publish the establishment of trophoblast organoid models (Turco et al., 2018). She described her lab's focus on using these models to understand how the uterine microenvironment influences human placental development. Recent work in her lab uses a high throughput image screening approach to identify maternally derived signals regulating extravillous trophoblast differentiation and invasion.

The second session of the meeting focused on pre- and post-implantation development using stem cells. Dr Thorold Theunissen from Washington University in St Louis, USA, described his earlier work in generating hTSCs from hPSCs. He showed that naïve but not primed hPSCs differentiate into hTSCs that exhibit a post-implantation cytotrophoblast state. These cells can also be used to grow trophoblast organoids with remarkable similarity to placenta-derived organoids. He gave a fascinating example of how hPSC-derived trophoblast organoids from female cells can be used to investigate random X-chromosome inactivation (Karvas et al., 2022). Finally, Dr Theunissen presented his group's latest findings on modelling human trophoblast expansion, diversification, and invasion using naïve hPSC-derived blastoids cultured to post-implantation stages on 3D matrices. Dr Yasuhiro Takashima's work at Kyoto University, Japan, focuses on human pre- and post- implantation stages using hPSCs. His lab induced hypoblast and trophectoderm from naïve hPSCs that recapitulate several key stages with pre-implantation embryos (Io et al., 2021; Okubo et al., 2024). Unlike primed hPSCs, which exhibit amnion-like features when differentiated into trophoblasts, naïve hPSCs were able to recreate the trophectoderm to cytotrophoblast transition. The inaccessibility to the early conceptus means that human post-implantation development remains a significant challenge to study *in vivo*. Non-human primates recapitulate several key features of human development that provides access to early post-implantation stages. Dr Thorsten Boroviak from the University of Cambridge describes his work using marmoset naïve PSCs to track the pre- to post-implantation transition of the trophoblast lineage. He demonstrated that the marmoset trophoblast stem cells (TSCs) undergo shallower implantation compared to humans, recapitulating the *in vivo* phenotype.

The third session continued the theme of comparative placentation. Dr Hongmei Wang (Chinese Academy of Sciences, China) described her lab's wide encompassing work on mammalian placentation using humans and non-human primate (cynomolgus) models. Her lab has produced some of the most detailed single cell atlases of human placentas across gestation (first, second and third trimester). One limitation of previous single cell RNA-sequencing datasets of the human placenta is that the syncytiotrophoblast population is under-represented due to the multinucleated nature of the syncytium, which is not amendable to standard single cell approaches. Her lab overcame this obstacle by using single nuclei RNA-sequencing to characterise the large multinucleated syncytium and demonstrated the heterogeneity between early versus late gestation syncytium (Wang et al., 2024). Current HEFA legislation limits the culture of human embryos beyond 14 days post-fertilization (dpf). To explore primate embryonic development beyond this period, Dr Wang's lab has used cynomolgus macaque embryos to optimise culture conditions to grow these embryos up to 25 dpf to the point of early neurulation (Zhai et al., 2023). The *in vitro* macaque embryos recapitulated the morphological, transcriptional, and epigenetic features of their *in vivo* counterparts. Dr Claudia Gerri's (Max Planck Institute of Molecular and Cell Genetics, Germany) lab aims to understand how the uterine microenvironment shapes the fetal-placental interface using a comparative embryology approach. In the first part of her talk, she demonstrated the role of oxygen and the extracellular matrix as two environmental stimuli, which shape human trophoblast development. In the second part, she presented work in understanding how and when diversity arises during development of various mammalian species. While the preimplantation embryos of humans, cows and mice appear very similar, the timing and localization of molecular markers differs across species (Gerri et al., 2023). Her latest work examines comparative placentation in pig, cat, cow and humans using trophoblast organoid models.

The fourth session turned the focus to mechanisms regulating early human development. Dr Martin Knofler from the Medical University in Vienna, Austria, provided an overview of his lab's work in understanding the key regulatory mechanisms governing trophoblast stemness and extravillous trophoblast differentiation. His lab's research focuses on Notch and Hippo signalling pathways, which are both critical for TSC expansion, and loss of their signalling components result in reduced TSC self-renewal and premature differentiation into syncytiotrophoblast (Dietrich et al., 2023; Meinhardt et al., 2020). Dr Laurent David (Nantes Université, France) presented his lab's multifaceted approach towards understanding human peri-implantation development. One of the major barriers to IVF success is that ∼60% of embryo transfers fail around implantation. By studying the molecular changes occurring during early human embryonic development, his lab has uncovered molecular signatures that identify when the embryos are ready to implant, and how embryos develop post-implantation. They then use blastoids to verify the function of molecular signatures which allow perturbation to understand function but also to improve IVF cultures. By employing a multi-omics approach (transcriptomic, proteomic, epigenomic and metabolomic) his lab demonstrated that TSCs are distinguished from PSCs by their low DNA methylation and high metabolic activity (Onfray et al., 2024).

The fifth and last session of the invited talks began with Dr Irene Zorzan (Babraham Institute, Cambridge, UK) presenting her work in understanding the epigenetic factors driving trophoblast specification. She showed exciting preliminary data of an *in vitro* co-culture system using endometrial stromal and epithelial cells with human blastoids to model human embryo implantation. Using this system, she identified an epigenetic factor which regulates the earliest stages of extravillous trophoblast differentiation. The last invited speaker, Dr Yonatan Stelzer from the Weizmann Institute, Israel, discussed his team's development of single-embryo single-cell time-resolved model of mouse gastrulation that maps continuous and parallel differentiation in embryonic and extraembryonic lineages (Mayshar et al., 2023). Using this approach, he demonstrated a dual role of BMP4 at different embryonic stages in both promoting as well as restricting lineage specification in time and space.

## Flash talks

Supplementing the sessions were ‘flash talks’, 3-min oral presentations selected from the submitted abstracts. These presentations were from ECRs showcasing their latest research. The goal of the flash talk was not to present detailed information but to entice attendees to the presenters' posters. A considerable proportion of the fourteen flash talks featured hTSC and organoid models reflecting the theme of the meeting.

## Evaluating current approaches to model the human placenta *in vitro* – a panel discussion

The last session of the meeting featured a panel discussion with all the invited speakers around the main theme of the meeting ‘emerging models of human placental development’. The discussion chair Dr Alex Beristain (University of British Columbia, Canada), started the session by highlighting the recent and vast developments in trophoblast research. The past 5-8 years have seen tremendous progress in *in vitro* models of the placenta, particularly in the areas of stem cell (trophoblast as well as PSC-derived) -based modelling of placental development and organoid systems to assess intercellular communication. The aim of the panel discussion was to reflect on this progress and evaluate the models in terms of their advantages and limitations, as well as the next steps towards improving these models.

The panel discussion centred on three key unresolved questions in the field.

**Question 1:** What placental cell type do current TSC and organoid-based systems model? This question was raised in part due to recent studies demonstrating the differences between 2D hTSCs versus 3D organoids. It appears that hTSC lines may represent a rare trophoblast population identified in single-cell atlases of first-trimester placentas, while organoids consist of a heterogeneous cell population with an expanded progenitor state (Shannon et al., 2024). Despite these differences, both cell platforms effectively model differentiation trajectories into both the syncytiotrophoblast and extravillous trophoblast lineage.

**Question 2:** Are TSCs derived from term chorionic villi actual TSCs? Recent studies indicate that trophoblast progenitor cells with the potential for long-term culture and 3D organoid growth can be derived from term placentas (Hawkins et al., 2024; Yang et al., 2022). Although term trophoblasts are capable of syncytialisation, it remains uncertain whether they also differentiate into extravillous trophoblasts. Furthermore, as Dr Okae noted in his presentation, term trophoblasts accumulate mutations and undergo epigenetic changes that restrict their biopotency.

**Question 3:** What key advancements and considerations are necessary for modelling trophoblast non-trophoblast interactions? One important concern when designing co-culture experiments is balancing the unique growth factor and supplement requirements for each cell type – finding the combination that supports two or more cell types therefore becomes challenging. Moreover, the physiological relevance of these supplements must be carefully evaluated, as some may not naturally occur within the *in vivo* tissue microenvironment. Recreating an intact syncytial barrier *in vitro* is another crucial, yet challenging, aspect essential for accurately modelling maternal-fetal transfer. The consensus among the panel suggests that the next frontier in trophoblast biology lies in establishing standardised methods to grow and model interactions between trophoblast and non-trophoblast cells effectively.

## Conclusions

The past 5-8 years have witnessed tremendous developments around *in vitro* placental modelling using hTSCs and organoids. This year's CTR meeting was an occasion to bring together the pioneers in this area to share their recent work. One of the challenges with this rapid progress is that given the vast number of different methodologies it may be difficult to understand what questions these models are able to address. Therefore, this meeting presented an opportunity to look back on this progress and evaluate the current models to assess what cell types they are modelling, their strengths, and their limitations. The take home messages from the end of the panel discussion were that: robust and detailed protocols are required to ensure reproducibility; that these models need to be benchmarked against *in vivo* tissues; and lastly the importance of sharing data (and code) especially from single cell atlases to allow for an integrated reference map to be built in the future.
